# A randomised controlled trial of an educational intervention to promote safe behaviours in petrochemical workers: a study protocol

**DOI:** 10.1186/s12889-019-7126-1

**Published:** 2019-06-18

**Authors:** Azita Zahiri Harsini, Fazlollah Ghofranipour, Hormoz Sanaeinasab, Farkhondeh Amin Shokravi

**Affiliations:** 1 Department of Health Education and Health Promotion, Faculty of Medical Sciences, Tarbiat Modares University, Tehran, Iran; 2 Health Research Center, Lifestyle institute, Baqiyatallah University of Medical Sciences, Tehran, Iran

**Keywords:** Safe behaviours, Educational intervention, Occupational hazards, Petrochemical workers

## Abstract

**Background:**

The worldwide concern about safety has created a need for new and effective strategies to improve safety in the workplace. Based on reported studies, approximately 90% of workplace accidents are due to unsafe behaviour and human error. Therefore, the most important strategy in reducing the rate of these accidents is training workers regarding safe behaviour and avoiding human error. There is limited research on understanding the barriers to promoting safe behaviour amongst petrochemical workers. This paper presents a protocol for an intervention study, using training sessions in combination with an educational software (application). The intervention aims to both promote workers’ safe behaviour and reduce the rate of occupational accidents.

**Methods:**

One hundred seventy-six workers will be recruited to this study from an Iranian petrochemical industry. The study is Mixed Methods Research (MMR) which will be carried out in two phases. In the first phase, using a qualitative approach, in-depth interviews will identify the causes of unsafe behaviour in the petrochemical industry. In the second phase, models of safe behaviour used in workplaces and the petrochemical industry will be investigated. The findings of the first phase will be matched with the constructs of these models to produce a well-suited conceptual model. Questionnaires and an educational intervention will be designed based on the results of the two phases. The workers will receive training interventions using direct methods, involving training sessions and workshops, and in an indirect method for which educational software will be designed. A randomised controlled trial (RCT) will assess comparability between the intervention group and the control group at baseline, after the intervention, and at a three-month follow up.

**Discussion:**

This research will provide a practical approach for promoting safe behaviours and reducing occupational hazards amongst industrial workers.

**Trial registration:**

Iranian Registry of Clinical Trials: IRCT20170515033981N2. Registered 19 June 2018.

## Background

Work-related accidents and injuries in industrial organisations have always been a major issue worldwide [1]. Despite all the positive changes and improvements in organisational structures and various technological developments in industrial settings, the number of serious injuries and occupational accidents has not decreased during the last decades [2, 3]. Moreover, as Sunindijo and Zou [4] argue, while the industry employs only about 7% of the workforce worldwide, it is responsible for 30–40% of fatalities. Based on statistics, 100,000 workers are killed on construction sites every year —one person every five minutes due to unsafe working conditions [5]. Annually, according to the International Labour Organization (ILO), it is estimated that more than 2.2 million workers die as a result of occupational accidents and work-related diseases. Across the globe, workers suffer approximately 270 million occupational accidents that lead to absences from work for three days or more, and fall victim to some 160 million incidents of work-related disease each year [6]. While cancers and cardiovascular diseases kill more workers than occupational injuries, the number of Years of Life Lost to work-related injuries is still significantly high as those injured were much younger than, for example, those who died from work-related cancers [7]. This fact highlights the importance of conducting research on safety in the industrial settings. Research in the area of occupational safety related to safe work behaviour has become prominent during the past decades [8]. Ensuring safe and healthy working conditions is an essential element of the quality of work life [9, 10]. Several researchers have attempted to determine the effects of workers’ occupational safe behaviour on work injuries across a range of industrial settings [11, 12]. Findings from a number of studies have indicated that unsafe work behaviour increases the probability of accidents and fatal injuries in organisations [13]. For example, Shin and colleagues [14] state that approximately 88% of workplace incidents in the construction industry are caused by unsafe behaviour, 10% by unsafe conditions and 2% by unforeseeable factors. Accordingly, all these reasons signify that safety has been considered an important issue in industrial settings. It is especially so in developing countries where workplace safety is a major area of concern because of the lack of safety regulations [15]. Therefore, identifying such unsafe acts and conditions is the first step in taking action to eliminate them, minimize the risk of work-related injuries or illnesses and prevent further accidents [16].

Behaviour-based safety (BBS) has been explored extensively across a variety of disciplines [13, 17–19]. Research focuses mainly on both person and situational aspects to explain safety in industrial environments and how individual and contextual factors interact to influence workplace safety [20]. The role of the construction project manager is crucial in increasing the level of safety performance [4]. Findings indicate that managers are responsible for the implementation of the safety policies of the company as well as the management of challenging tasks which require them to have sufficient skills [21]. Thus, managers need the ability to influence workers to adopt positive behavioural attitudes and intentions towards work safety [22]. They also should report and investigate work-related accidents and near-misses to identify the root causes, and receive feedback from worker [23, 24]. Safety climate culture is generally accepted as another crucial indicator for enhanced safety in organisations [25, 26]. A number of notable safety climate studies provide empirical evidence to support a positive link between safety climate and safe work behaviour [27–29]. Zohar defined safety climate as the sum of shared perceptions among employees with regard to safety procedures, policies, and practices in the work environment [30]. The development of a safety climate could be related to the elimination of unsafe acts and conditions and could lead to the prevention of occupational accidents and safety performance improvement [31, 32]. Another major contribution of the origins of safety performance is workers’ knowledge of the safety procedures to manage safety risks [33]. It is predicted that workers tend to make mistakes because they have lack of understanding on how to follow organisational safety procedures and regulations, are unaware of the potential hazards in their work environment, and have negative attitudes towards safety [34]. It is therefore expected that a higher level of knowledge acts as a mechanism that increases workers’ behavioural intentions to engage in safe work behaviours [35, 36].

Based on the published data from Iran, the trend of occupational accidents and work-related diseases is the same as the rest of the world [37]. According to the Iranian Ministry of Labour, work-related injuries are mainly caused by unsafe work conditions as well as some individual characteristics [38]. Petrochemical industries have been classified as a high-risk industry to the potential for serious occupational accident consequences and severe effects to the environment [39]. Iran exports its petrochemical products to several dozen countries in different parts of the world and this has positioned Iran as the second largest producer and exporter of petrochemicals in the Middle East [40]. Norozi and colleagues reported that in Iranian petrochemical companies, more than 198 fatal injuries occurred over the last decade [41].

Research has shown that training of workers plays the most vital role in safety at work [42]. There is evidence that safety training is considered as a safety improvement intervention to reduce accident rates [43]. First aid training and safety training is the enhancement of safety knowledge that should be known by workers to guide them in how to improve their compliance with health and safety requirements. Managers must provide direct safety practices for workers to understand safety procedures [43, 44]. The importance of safety training has also been emphasised by Loosemore and Malouf [45] who found that the knowledge acquired through many training programs often made workers more aware that their own behaviour and was an important factor in the avoidance of work-related injuries and illnesses, and also appeared to reduce workers’ willingness to accept prevailing levels of occupational safety risk on industrial sites.

By considering the increasing number of recent accidents in Iranian petrochemical companies, qualitative research enables us to more fully understand workers’ perceptions of the factors influencing safe behaviour and how to promote and maintain such behaviour which will, in turn, prevent injuries and workplace absenteeism. This paper presents the design of a study for the educational intervention in an Iranian petrochemical company, in which multiple forms of data (qualitative and quantitative) are incorporated and evaluates the intervention’s effectiveness. We hypothesize that this intervention will be effective in terms of promoting safe work behaviour and will also reduce work-related incidents in the intervention group at the end of the study, compared to the control non-intervention group.

### Aims and research question

The overall aim of this study is to design and evaluate an educational intervention to promote workers’ safe work behaviour. The specific objectives include:

1. To design/ develop a conceptual model of safe work behaviour based on qualitative data;
2. To design/ develop a questionnaire of safe behaviour based on the constructs of the conceptual model;
3. To design an educational intervention based on the findings of the first two phases of the study to promote and maintain safe work behaviour.

The main research question is to determine whether the educational intervention leads to the promotion and maintenance of safe behaviour in the intervention group compared to the control group.

## Methods

This study will be conducted and reported on the basis of Consolidated Standards of Reporting Trials (CONSORT) 2010 statement [46].

### Study design

**Fig. 1:**
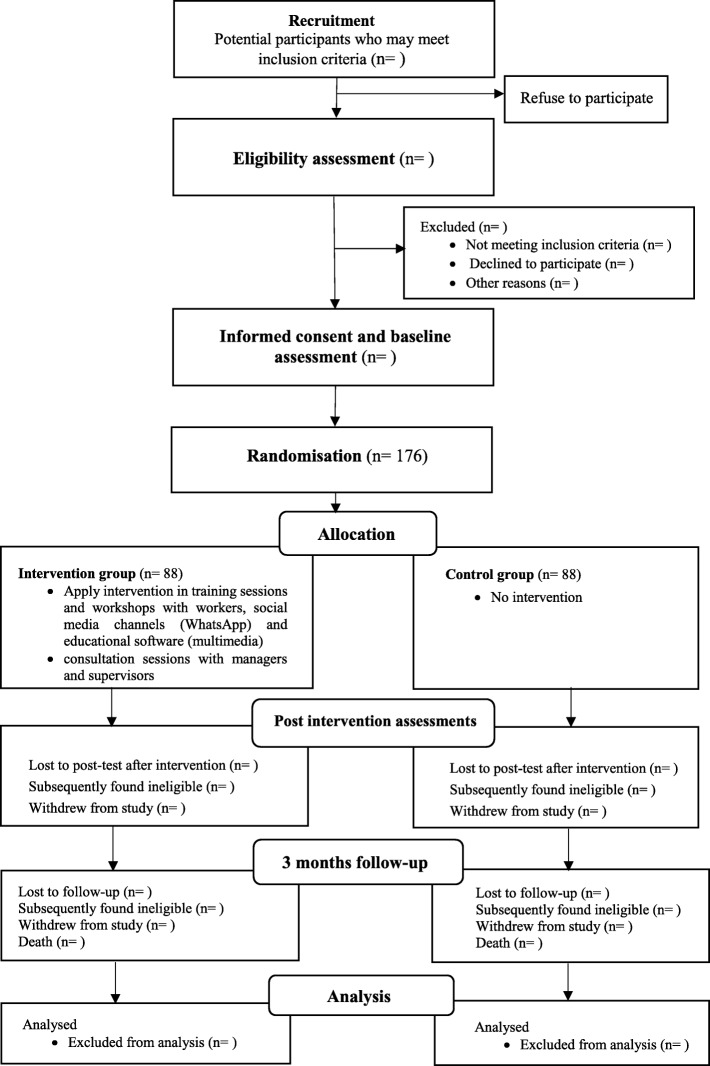
CONSORT flow diagram of the study design. This study will be conducted and reported on the basis of Consolidated Standards of Reporting Trials (CONSORT) 2010 statement. The figure shows the flow diagram of randomized controlled protocol

The educational intervention will be evaluated using a randomised controlled trial. The Medical Research Ethics Committee of Tarbiat Modares University in Iran approved the study protocol (Approval ID: IR.TMU.REC.1395.503). All participants will provide written consent to participate in the study, will be advised that data are going to be anonymised, securely stored, and analysed for publication. They will be advised that participation will be voluntary, and they are free to leave the study at any time. A flow diagram of randomized controlled protocol is shown in Fig. 1.

### Study sample

#### Participant recruitment and eligibility criteria

A total of 176 workers will be recruited from an Iranian petrochemical industry. Since we seek to obtain a broad cross-section of worker opinions and experiences, we will use a combination of purposive sampling and maximum variation sampling strategies. Staff from the company’s Safety, Health and Environment unit, who are not part of the research team, will invite workers, supervisors, and safety managers from various occupational groups working in the operations department and the maintenance and repair department who have experienced accidents and injuries or have witnessed colleagues’ accidents to participate in the study (purposive sampling). Workers are eligible to participate if they have (1) work experience in the petrochemical industry for at least two years, and (2) participants’ motivation to recount their experiences to the researcher. The exclusion criteria include (1) the reluctance of participants to continue to participate in the study, and (2) the lack of regular attendance at training sessions. During the interviews, respondents will identify employees who have information about workplace accidents in the company and are key informants (snowball sampling). These employees will also be invited to participate in the study. Before the start of each interview, a member of the safety staff will introduce the participant to the first author, who will provide clear verbal information about the study.

#### Setting and sample size

The research setting is a petrochemical industry in Iran. The study population consists of workers of this Iranian petrochemical company. Since this study aims to promote workers’ safe behaviour, as reported by Waters and Duncan [47] (a similar study), the effect of the behavioural safety program interventions that is considered as the dependent variable in this study has been 40%, if it is assumed that environmental factors and non-compliance by control group participants account for about 50% of this effect, namely 20%, for each group, based on the following formula [48] and with 95% confidence interval and 80% power of a test, 78 participants will be required.. The researcher will hypothesize a 10% attrition rate (in both groups). In order to ensure that the total sample size is available, the researcher will need to recruit more participants to allow for attrition. Therefore, with the anticipated attrition rate, the researcher should enrol 176 participants. The participants will be randomly allocated to the intervention group (*n* = 88) or the control group (*n* = 88).

$$ {\displaystyle \begin{array}{c}n=\frac{{\left({Z}_{1-\alpha 2}+{Z}_{1-\beta}\right)}^2\left({P}_1\left(1-{P}_1\right)+{P}_2\left(1-{P}_2\right)\right)}{{\left({P}_1-{P}_2\right)}^2}\\ {}=\frac{{\left(1.96+0.84\right)}^2\left(0.4(0.6)+(0.2)(0.8)\right)}{{\left(0.4-0.2\right)}^2}=78.4\end{array}} $$

### Measure

This study is Mixed Methods Research (MMR), in which the research methodology will be presented as follows:

1. Conducting qualitative research;
2. Conducting a literature search to identify theoretical models that have been used to explain and predict safe behaviour in the workplace, both in the petrochemical industry and more generally, and selecting a theoretical model that provides a suitable organising structure for each of the phases of research – quantitative phase, survey, and RCT;
3. Conducting a randomised controlled trial (RCT) to assess the effectiveness of the intervention.

### Phase one: qualitative exploration

#### Approach to interviewing

Semi-structured interviews will be conducted to gain in-depth understanding of factors associated with unsafe work behaviour in the company. Probing questions will be used when answers are vague or ambiguous or to obtain more specific or in-depth information. Conducting the interviews and analysis of the qualitative data will occur through an iterative process, such that data from earlier interviews will be allowed to influence the content of later interviews. All respondents will be asked identical questions in the same sequence, but the interviewer will probe inductively on key responses. Sometimes the respondents will provide information on new areas and these will be included in subsequent interviews. This process enables the researcher to gain deeper insight into the data, while continuing the data collection [49]. Data will be collected until no more new themes emerge from the data and the interviewer is confident that data saturation has been achieved [50, 51].

#### Data collection

The interviews will be conducted in the period May – July 2017 at mutually convenient and private areas at the participants’ workplaces. The interview questions will be classified into three categories:

1. What are your individual feelings or perceptions of being safe at work?
2. Have you experienced workplace accidents directly yourself or have you witnessed accidents by your colleagues in the workplace?
3. What are the main factors contributing to the occurrence of workplace accidents?

The interviews will be audiotaped and a summary of the key issues in each interview will be then sent to each participant to ensure that the researcher will have accurately interpreted that participant’s comments (a ‘member check’) [52]. All interviews will be then transcribed in Persian and translated into English for qualitative analysis. Any identifying information in the transcripts will be removed prior to data coding.

#### Data analysis

Conventional content analysis will be used to interpret the content of text data through a systematic classification process involving coding and identifying themes [53]. Open coding will be carried out to allow codes to emerge from the qualitative data and avoid codes based on predispositions of the authors. Immersion in the data is an important first stage in the analysis process during which transcripts will be read and re-read many times to become completely familiar with the data. Repeated reading and re-reading of transcripts without coding helps identify emergent themes from the data without losing the connections between key concepts and their context. Content analysis will be performed using MAXQDA (Ver. 2018) software to facilitate and document the coding process and retrieve codes afterwards. It should be noted that while software can assist researchers to organise qualitative data, computer software for qualitative analysis does not analyse data and the researcher makes decisions about coding participants’ responses, and the relationships between codes, coding categories and broader themes. MAXQDA allows the researcher to upload raw data, such as transcribed interviews, that then can be coded and cross-referenced in ways that facilitate organising the data for easy retrieval.

This study will employ the approach to qualitative content analysis described by Graneheim and Lundman [54]. This approach consists of the following elements: units of analysis, meaning units, condensation of meaning units, and development of codes, categories and themes. One of the most basic decisions when using content analysis is selecting the unit of analysis. Whole interviews or observational protocols are considered to be units of analysis. In the second step, the interview text is divided into smaller units called meaning units. A meaning unit — which could be words, sentences or paragraphs — contains aspects, words or statements that relate to the same central meaning. In the third analysis step, condensation, units are shortened while still preserving their core meaning. In the fourth step, codes are developed as descriptive labels for the meaning units. They are tools to help researchers reflect on the data in new and different ways. The fifth step is to sort codes into categories that answer the question, “What?”. In other words, a category is formed by grouping together those codes that are associated with each other through their content or context and belong together. A theme can be seen as expressing the underlying meanings together in two or more categories. The final step of data analysis is the creation of themes. A theme answers the question, “How?”. Therefore, theme names include verbs, adverbs and adjectives and are very descriptive.

### Phase two: questionnaire survey

A variety of conceptual models of workplace safety have been developed to help organise research findings and guide the development of occupational safety programs [55–57]. These models are useful because they highlight the importance of workers’ job characteristics, the job tasks being performed and the organisational structure. These models have been applied to raise workers’ awareness of occupational hazards and encourage them to adopt safe behaviour [58, 59].

In present study, theoretical models which have been applied to explain and predict safe behaviour in industrial settings, more generally and in parallel, and theoretical models that have been developed or applied in the petrochemical companies will be investigated. Findings that emerge from the qualitative phase will then be employed to identify the best model fits the data. In regard to the model components, questionnaires will be designed/ developed to conduct a pre-test survey. Based on information obtained from this, educational needs assessment will be conducted and then an educational intervention will be designed in order to train workers.

#### Questionnaires

The questionnaire will consist of two parts. The first part will cover social demographics and the second part will include the constructs of the conceptual model. Data will be collected at baseline, after the intervention, and after three months for workers from the intervention group and the control group. The questionnaires will be anonymous to promote participation and workers’ confidence in the intervention. All the participants who will be doing measurements will be blinded to the assigned groups of the study.

### Phase three: randomised controlled trial (RCT)

The randomised controlled trials (RCTs) is the basic methodological paradigm for the evaluation of public health interventions. RCTs aim to generate that the assignment of study subjects to the intervention or the control group is exclusively due to chance, thus avoiding effects of confounding and selection biases. The availability of a control group makes it possible to distinguish between epidemiological and/or statistical associations and cause-effect relations [60]. The RCT design is a significant strength of this study, including qualitative and quantitative measures to assess the effectiveness of the intervention.

#### Intervention

The intervention will include multiple components associated with information obtained from pre-test stage and constructs of the conceptual model. Participants will attend eight weeks of biweekly 90-min intervention sessions. Each session will consist of two parts, a 60-min interactive workshop followed by a 30-min group activity. There will be different themes for each of the workshops, which will start with a presentation to highlight the important factors, and effective strategies for changing unsafe behaviour to overcome barriers they may have encountered and discussions between workers and the researcher. Then, each workshop will be followed by group activities. All video and written teaching materials for these sessions will be prepared by the research team. One important goal of the workshops is to teach workers how to identify workplace hazards, to promote their own safe behaviour and maintain it. In the second part of the intervention, texts and messages related to different aspects of workplace safety will be sent to the participants in the intervention group via WhatsApp. Apart from the workshops, there will be two consultation sessions with managers and supervisors during the intervention period. These sessions will prompt them to be fully committed to safety issues so that top prioritisation of safety will be widely accepted. Finally, educational software (multimedia) will be designed. Participants allocated to the control group will be asked to attend measurement sessions, but they will only receive the intervention six months later (i.e., after the follow-up time point).

Analyses will be conducted using SPSS Ver.24 and alpha levels will be set at *p* > 0.05. Descriptive statistics will be applied to describe the study population at baseline. The categorical variables will be analysed using frequency distribution description and centralised trend description. The continuous variable will be analysed using means ± standard deviations. The difference of characteristics of participants will be compared by t test or chi-square 2 test or non-parametric equivalent test. Effectiveness of the intervention will be measured by the differences between the study groups (intervention group and control group). To provide a more comprehensive analysis of the theoretical relationships among the factors in the conceptual model, path analysis techniques will be applied with AMOS Ver.24. Qualitative data from semi-structured interviews will be processed using MAXQDA (Ver. 2018).

## Discussion

This study is designed to promote safe work behaviour among petrochemical workers. To the best of our knowledge, there are no qualitative studies that have examined workers’ perceptions about factors affecting safe work behaviour in the petrochemical industry that could be used to inform the design of educational interventions in the published literature. The aim of this study is to address an important gap in the evidence on the impact of educational interventions aimed at improving safety-related behaviours may be delivered to workers, safety professionals or managers of the organisational settings using an RCT design. The mixed methods RCT design is a significant strength of this study which would often not be considered for evaluating the impact of educational programs in industrial research to date. RCTs are considered to be the “gold standard” with the highest internal validity and least amount of bias in the clinical and health care interventions, but these are less commonly utilised to evaluate the effectiveness of workplace interventions. As this evaluation illustrates, trials are relevant to workplace interventions and it is feasible to conduct an RCT in a work environment such as the petrochemical industry. Our study has also potential limitations that we need to consider. We will be conducting this RCT among workers in a petrochemical company, which means all participants are male. They may not be representative of the entire population, especially for workers in other organisations in Iran. A further limitation of this study is the use of self-report measures. Memory error, nondisclosure, social desirability concerns, and intentional misrepresentation may affect reporting accuracy [61]. To reduce the chance of this occurring, standardised tools validated for use with vulnerable populations will be used where possible [62]. Maximized participation will be regularly reinforced by providing bonuses. We anticipate that this study will assist in prioritising safety regardless of administrative pressure. Therefore, if shown effective, these training sessions could be organised and delivered by trained trainers in the future. Consequently, this intervention could be scaled to a larger number of Iranian industries in the future and could impact a broader population of workers.

## Data Availability

Not applicable – this is a research protocol and does not contain any data.

## Abbreviations

BBS: Behaviour-based safety
ILO: International Labour Organization
MMR: Mixed Methods Research
RCT: Randomised controlled trial

## References

[1] Nenonen S: Implementation of safety management in outsourced services in the manufacturing industry. Tampereen teknillinen yliopisto Julkaisu-Tampere University of Technology Publication; 1023 2012.

[2] Tappura S, Nenonen N, Kivistö-Rahnasto J (2017). Managers’ viewpoint on factors influencing their commitment to safety: an empirical investigation in five Finnish industrial organisations. Saf Sci.

[3] Newaz MT, Davis P, Jefferies M, Pillay M (2019). The psychological contract: a missing link between safety climate and safety behaviour on construction sites. Saf Sci.

[4] Sunindijo RY, Zou PX (2012). How project manager's skills may influence the development of safety climate in construction projects. Int J Proj Org Manag.

[5] Murie F (2007). Building safety—an international perspective. Int J Occup Environ Health.

[6] ILO (2005). World day for safety and health at work 2005: a background paper.

[7] Takala J, Hämäläinen P, Nenonen N, Takahashi K, Chimed-Ochir O, Rantanen J (2017). Comparative analysis of the burden of injury and illness at work in selected countries and regions. Cent Eur J Occup Environ Med.

[8] Zaira MM, Hadikusumo BH (2017). Structural equation model of integrated safety intervention practices affecting the safety behaviour of workers in the construction industry. Saf Sci.

[9] Fugas CS, Silva SA, Meliá JL (2012). Another look at safety climate and safety behavior: deepening the cognitive and social mediator mechanisms. Accid Anal Prev.

[10] Amponsah-Tawaih K, Adu MA (2016). Work pressure and safety behaviors among health workers in Ghana: the moderating role of management commitment to safety. Saf Health Work.

[11] Neal A, Griffin MA (2006). A study of the lagged relationships among safety climate, safety motivation, safety behavior, and accidents at the individual and group levels. J Appl Psychol.

[12] Varonen U, Mattila M (2000). The safety climate and its relationship to safety practices, safety of the work environment and occupational accidents in eight wood-processing companies. Accid Anal Prev.

[13] Choudhry RM (2014). Behavior-based safety on construction sites: a case study. Accid Anal Prev.

[14] Shin D-P, Gwak H-S, Lee D-E (2015). Modeling the predictors of safety behavior in construction workers. Int J Occup Saf Ergon.

[15] Mohamed S, Ali TH, Tam W (2009). National culture and safe work behaviour of construction workers in Pakistan. Saf Sci.

[16] Jeelani I, Han K, Albert A (2018). Automating and scaling personalized safety training using eye-tracking data. Autom Constr.

[17] Eid J, Mearns K, Larsson G, Laberg JC, Johnsen BH (2012). Leadership, psychological capital and safety research: conceptual issues and future research questions. Saf Sci.

[18] Hedlund A, Gummesson K, Rydell A, Andersson M (2016). Safety motivation at work: evaluation of changes from six interventions. Saf Sci.

[19] Roberts KH, Bea R (2001). Must accidents happen? Lessons from high-reliability organizations. Acad Manag Perspect.

[20] Christian MS, Bradley JC, Wallace JC, Burke MJ (2009). Workplace safety: a meta-analysis of the roles of person and situation factors. J Appl Psychol.

[21] Sunindijo RY, Zou PX (2011). CHPT construct: essential skills for construction project managers. Int J Proj Org Manag.

[22] Hald KS (2018). Social influence and safe behavior in manufacturing. Saf Sci.

[23] Pagell M, Gobeli D (2009). How plant managers' experiences and attitudes toward sustainability relate to operational performance. Prod Oper Manag.

[24] Pagell M, Johnston D, Veltri A, Klassen R, Biehl M (2014). Is safe production an oxymoron?. Prod Oper Manag.

[25] Liu X, Huang G, Huang H, Wang S, Xiao Y, Chen W (2015). Safety climate, safety behavior, and worker injuries in the Chinese manufacturing industry. Saf Sci.

[26] DeJoy DM, Della LJ, Vandenberg RJ, Wilson MG (2010). Making work safer: testing a model of social exchange and safety management. J Saf Res.

[27] Siu Oi-ling, Phillips David R, Leung Tat-wing (2004). Safety climate and safety performance among construction workers in Hong Kong. Accident Analysis & Prevention.

[28] Dedobbeleer N, Béland F (1991). A safety climate measure for construction sites. J Saf Res.

[29] Zhang RP, Lingard H, Nevin S (2015). Development and validation of a multilevel safety climate measurement tool in the construction industry. Constr Manag Econ.

[30] Zohar D (2010). Thirty years of safety climate research: reflections and future directions. Accid Anal Prev.

[31] Gillen M, Baltz D, Gassel M, Kirsch L, Vaccaro D (2002). Perceived safety climate, job demands, and coworker support among union and nonunion injured construction workers. J Saf Res.

[32] Pousette A, Larsson S, Törner M (2008). Safety climate cross-validation, strength and prediction of safety behaviour. Saf Sci.

[33] Chatzoudes D, Chatzoglou P, Vraimaki E (2015). The central role of knowledge management in business operations: developing a new conceptual framework. Bus Process Manag J.

[34] Lingard H, Rowlinson S. Occupational health and safety in construction project management. New York: Spon Press; 2005.

[35] Mowday RT, Porter LW, Steers RM. Employee-organization linkages: the psychology of commitment, absenteeism, and turnover. New York: Academic Press; 1982.

[36] Kanter Rosabeth Moss (1968). Commitment and Social Organization: A Study of Commitment Mechanisms in Utopian Communities. American Sociological Review.

[37] Kamalvandi M, Mohammadfam I, Farhadi R, Jalilian M, Kurd N (2017). Evaluation of work-related accidents among Hamadan construction workers. J Basic Res Med Sci.

[38] Rahmani A, Khadem M, Madreseh E, Aghaei H-A, Raei M, Karchani M (2013). Descriptive study of occupational accidents and their causes among electricity distribution company workers at an eight-year period in Iran. Saf Health Work.

[39] Jaderi F, Ibrahim ZZ, Zahiri MR (2019). Criticality analysis of petrochemical assets using risk based maintenance and the fuzzy inference system. Process Saf Environ Prot.

[40] Gholami PS, Nassiri P, Yarahmadi R, Hamidi A, Mirkazemi R (2015). Assessment of health safety and environment management system function in contracting companies of one of the petro-chemistry industries in Iran, a case study. Saf Sci.

[41] NOROZI M, Jahangiri M, Choobineh A, Narimannejad A (2013). Feasibility study of implementing process safety management (PSM) requirements in an Iranian petrochemical company. Int J Occup Hyg.

[42] Hinze J, Gambatese J (2003). Factors that influence safety performance of specialty contractors. J Constr Eng Manag.

[43] Tam C, Zeng S, Deng Z (2004). Identifying elements of poor construction safety management in China. Saf Sci.

[44] Shin M, Lee H-S, Park M, Moon M, Han S (2014). A system dynamics approach for modeling construction workers’ safety attitudes and behaviors. Accid Anal Prev.

[45] Loosemore M, Malouf N (2019). Safety training and positive safety attitude formation in the Australian construction industry. Saf Sci.

[46] Schulz KF, Altman DG, Moher D (2010). CONSORT 2010 statement: updated guidelines for reporting parallel group randomised trials. BMC Med.

[47] Waters Robert M., Duncan Michael (2001). Behavioral Safety Programs in the Department of Energy. Proceedings of the Human Factors and Ergonomics Society Annual Meeting.

[48] Pocock SJ. Clinical trials: a practical approach: Wiley; 2013.

[49] Wesselman LM, Schild A-K, Coll-Padros N, van der Borg WE, Meurs JH, Hooghiemstra AM, Slot RE, Sannemann L, Rami L, Molinuevo JL (2018). Wishes and preferences for an online lifestyle program for brain health—a mixed methods study. Alzheimers Dement.

[50] Guest G, Bunce A, Johnson L (2006). How many interviews are enough? An experiment with data saturation and variability. Field Methods.

[51] Saunders B, Sim J, Kingstone T, Baker S, Waterfield J, Bartlam B, Burroughs H, Jinks C (2018). Saturation in qualitative research: exploring its conceptualization and operationalization. Qual Quant.

[52] Krefting L (1991). Rigor in qualitative research: the assessment of trustworthiness. Am J Occup Ther.

[53] Hsieh H-F, Shannon SE (2005). Three approaches to qualitative content analysis. Qual Health Res.

[54] Graneheim UH, Lundman B (2004). Qualitative content analysis in nursing research: concepts, procedures and measures to achieve trustworthiness. Nurse Educ Today.

[55] Swuste P, Van Gulijk C, Zwaard W, Oostendorp Y (2014). Occupational safety theories, models and metaphors in the three decades since world war II, in the United States, Britain and the Netherlands: a literature review. Saf Sci.

[56] Cohen A, Smith MJ, Anger W (1979). Self-protective measures against workplace hazards. J Saf Res.

[57] DeJoy DM, Southern DJ (1993). An integrative perspective on work-site health promotion. J Occup Med.

[58] Swuste P, van Gulijk C, Zwaard W (2010). Safety metaphors and theories, a review of the occupational safety literature of the US, UK and the Netherlands, till the first part of the 20th century. Saf Sci.

[59] Smith MJ, Beringer DB (1987). Human factors in occupational injury evaluation and control.

[60] Bonell C, Fletcher A, Morton M, Lorenc T, Moore L (2012). Realist randomised controlled trials: a new approach to evaluating complex public health interventions. Soc Sci Med.

[61] Tsemberis S, Gulcur L, Nakae M (2004). Housing first, consumer choice, and harm reduction for homeless individuals with a dual diagnosis. Am J Public Health.

[62] Bhandari A, Wagner T (2006). Self-reported utilization of health care services: improving measurement and accuracy. Med Care Res Rev.

